# A clinical study evaluating the combination of LISA and SNIPPV for the treatment of respiratory distress syndrome in preterm infants

**DOI:** 10.1038/s41598-023-50303-0

**Published:** 2024-01-16

**Authors:** Dhivya Lakshmi Permall, Yuhan Zhang, Hanyue Li, Yafei Guan, Xiaoqing Chen

**Affiliations:** 1 Nanjing Medical University, Nanjing, Jiangsu China; 2 Department of Pediatrics, The First Affiliated Hospital of Nanjing Medical University, Nanjing, Jiangsu China

**Keywords:** Paediatric research, Clinical trial design

## Abstract

To compare the therapeutic effect of less invasive surfactant administration (LISA) followed by synchronized nasal intermittent positive pressure ventilation (SNIPPV) and traditional intubate-Surfactant-Extubate (InSurE) strategy for the treatment of neonatal respiratory distress syndrome (NRDS). A single-center, non-randomized and single- blinded study Tertiary neonatal intensive care unit 89 infants enrolled were preterm with gestational age < 36^6/7^ weeks and clinically diagnosed with neonatal RDS (NRDS) Interventions: 32 infants were assigned to the LISA + SNIPPV group and 57 infants to the InSurE + nCPAP group. No statistically significant differences were noted in the baseline characteristics of the enrolled infants. A lower proportion of infants developed BPD in the LISA + SNIPPV group compared to the InSurE + CPAP group [10 (31.25%) vs. 21 (36.84%), P > 0.05]; however, there was no statistically significant difference. The number needed to treat (NNT) with LISA + SNIPPV to prevent BPD development is 18. The mortality rate was not significant between our study arms [1 (3.13%) vs 2 (3.51%), P > 0.05]. There were no statistically significant differences in the durations (days) of MV [(12.18 ± 13.89) vs. (11.35 ± 11.61), P > 0.05], oxygen therapy [(35.03 ± 19.13) vs. (39.75 ± 17.91), P > 0.05] and re-intubation rates [(0.19 ± 0.40) vs. (0.21 ± 0.45), P > 0.05] between the two study groups. In terms of complications, the incidence of patent ductus arteriosus (PDA) [24 (75.00%) vs. 27 (47.37%), P < 0.05] was higher and a lower rate of disturbed liver function [1 (3.23%) vs. 19 (33.33%), P < 0.05] were observed in the LISA + SNIPPV group. Acid–base imbalances were reportedly significantly higher in the InSurE group (P < 0.05). No significant differences in other complications were noted. In the interventional group, FiO2 requirements were significantly lower up until the 3rd week of treatment [FiO2 at day 0, (30.75 ± 4.78) vs. (34.66 ± 9.83), P < 0.05; FiO2 at day 21, (25.32 ± 3.74) vs. (29.11 ± 8.17), P < 0.05], as was RSS on days 2 [(0.77 ± 0.38) vs. (1.94 ± 0.75), P < 0.05] and 3 [(0.66 ± 0.33) vs. (1.89 ± 0.82), P < 0.05] after treatment. Additionally, infants in the standard group had a significantly prolonged hospital stay (days) [(45.97 ± 16.93) vs. (54.40 ± 16.26), P < 0.05]. The combination of LISA and SNIPPV for NRDS can potentially lower the rate of BPD, FiO2 demand and shorten the length of hospitalization.

## Introduction

To minimize neonatal lung injury, pulmonary surfactant administration has also evolved towards less invasive strategies over the years. Although the intubate-surfactant-extubate (InSurE) strategy has gained popularity since its initial description two decades ago and is currently the mainstay of treatment for RDS, it is still associated with short-term positive pressure ventilation. With advances in neonatal care and increasing data to support less invasive techniques of surfactant administration, less invasive surfactant administration (LISA) or minimally-invasive surfactant therapy (MIST) techniques are emerging. These revolutionary techniques have alleviated the reliance on MV and instead use non-invasive ventilatory modes to support spontaneously breathing infants during the procedure. Our study combines the use of LISA and synchronized nasal intermittent positive pressure ventilation (SNIPPV) for more advantageous outcomes in our study population.

With the increasing survival rate of extremely preterm infants due to advanced care in neonatal intensive care units (NICUs) and delivery room interventions for neonatal pulmonary conditions, a parallel increase in the incidence of BPD has been noted^1^. Hence, investigation to acquire the optimal and least invasive therapeutic plan for RDS is ongoing.

In a randomized controlled trial (RCT) aiming to eliminate the detrimental effects of long-term MV, Verder et al. were the first to analyze the InSurE (intubation-surfactant-extubation) method followed by nasal continuous positive airway pressure (nCPAP) in RDS infants^2^. They found a reduced need for MV and better oxygenation in the InSurE group, compared to the nCPAP only group. Dani et al. also reported a significant reduction in the duration of oxygen therapy, MV, hospital stay and decreased requirement for repeated surfactant doses in their surfactant-nCPAP compared to the surfactant-MV group^3^. The advantageous effects of InSurE were once again described in a retrospective, bi-center study in Stockholm, where a 50% reduction in the need for MV was observed after InSurE implementation at one of the centers^4^. Further reports also found InSurE to be associated with reduced need for subsequent MV, lower incidence of air leak syndromes (pulmonary interstitial emphysema and pneumothorax) and BPD^5,6^. Nonetheless, the adverse effects of premedication associated with the InSurE procedure cannot be neglected^7^. InSurE failure has been well-documented and although remifentanil and propofol are promising drugs for premedication, there is currently no consensus to guide their use during the InSurE procedure in neonates^7^. A further hindrance associated with InSurE is the acquisition of prompt intubation skills, which are of paramount importance. With the widespread use of nCPAP in recent years, it is nowadays harder for the junior pediatric personnel to gain adequate experience with newborn intubation. A study by O’Donnell et al. observed successful intubation only about 60% of the time^8^.

Hence, in 2007, Kribs et al. first reported the feasibility of a novel, less invasive surfactant administration (LISA) method, whereby direct laryngoscopy and Magill forceps are used to pass a thin catheter (feeding tube) into the trachea for surfactant administration in spontaneously breathing infants maintained on CPAP^9^. Similarly, in 2011, a different adaptation of LISA was performed by Dargaville et al. in which they used a 16 gauge vascular catheter for tracheal catheterization and the procedure was termed minimally-invasive surfactant therapy (MIST). The latter was also found to be an effective approach for surfactant delivery^10^. The goal of our research was to further optimize the strategy of surfactant administration via tracheal instillation and strive to find the most suitable post-extubation non-invasive ventilation (NIV) strategy so as to avoid InSurE and CPAP failure. Studies have previously demonstrated the superiority of nasal intermittent positive pressure ventilation (NIPPV) over nCPAP in premature infants in terms of lesser need for intubation and prevention of respiratory failure^11,12^. NIPPV has also been found to significantly reduce post-extubation failure compared to nCPAP^12^. As the beneficial roles of synchronized ventilation in premature infants have also been well-documented, synchronized NIPPV (SNIPPV) mode was applied to LISA-treated infants to maximize the benefits for our infants. SNIPPV improves thoraco-abdominal synchrony, decreases inspiratory effort and need for intubation and has a favorable effect on surfactant distribution in the lungs^12–15^. SNIPPV has also been found to be superior to NIPPV and nCPAP as it lowers the overall incidences of central apnea, desaturations and bradycardia^16^. Lower incidences of BPD and air leaks have also been reported. Taken together, we aimed to further assess the application of the promising LISA technique in combination with the more advantageous SNIPPV non-invasive respiratory support in the treatment of RDS in premature and low-birth weight infants.

## Methods

### Study design

**Figure 1:**
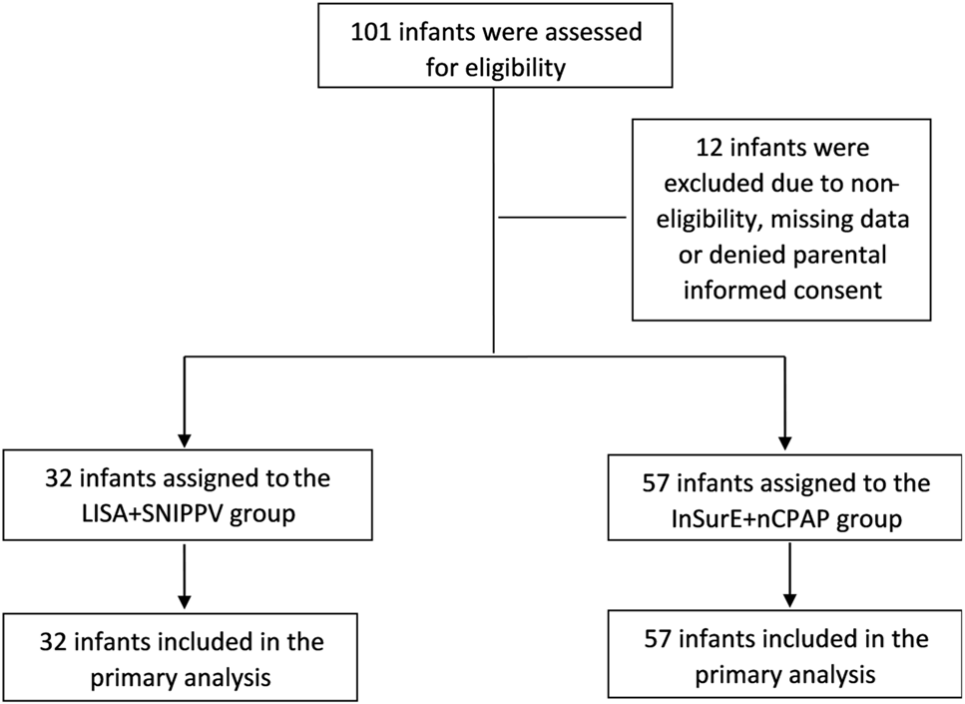
CONSORT diagram.

This was a single-center, non-randomized and single-blinded study. Masking of the treatment allocation was not possible but data documentation and analysis were performed in a blinded manner. The study was conducted in the NICU of The First Affiliated Hospital of Nanjing Medical University, Nanjing, China, between August 2018 and December 2019. A total of 89 infants were recruited in our study, 32 infants were assigned to the experimental LISA + SNIPPV group and 57 infants were assigned to the traditional InSurE + nCPAP group (Fig. 1). The decision for treatment allocation was based on a mutual decision involving the neonatologists and the parents. The study was approved by the ethics committee and institutional review board of The First Affiliated Hospital of Nanjing Medical University (2018-SR-161). And we have registered the experiment with ClinicalTrails.gov, the registration number:NCT03989960(Date:18/06/2019). This study was conducted in accordance with the Declaration of Helsinki. The study did not include adults, only premature infants. Written informed consent was obtained from the parents of each infant participating in the study.

### Management of premature infants

Delayed cord ligation was not performed in asphyxiated neonates, and the resuscitation steps were performed according to the Chinese resuscitation guidelines (revised in 2016 and 2021)^17,18^. The radiant warming table was preheated in advance, and the temperature of the radiant warming table was set at 32 ~ 34 °C for term infants, and according to its neutral temperature for preterm infants. All infants were required to have their heads dried and kept warm. Full-term infants were wrapped in preheated towels, dried and placed on the radiant warming table. For resuscitation of preterm infants < 32 weeks of gestational age and/or birth weight < 1500 g, the body and limbs below the head were wrapped in a clean plastic film/bag or covered with plastic wrap and placed on the radiant warming table, and initial resuscitation was continued in the correct position.

### Inclusion and exclusion criteria

The inclusion criteria for our study were:

1. Premature infants with birth weight < 2500 g and gestational age (GA) < 36^6/7^ weeks,
2. High-risk premature infants with early symptoms of RDS or infants clinically diagnosed as RDS,
3. Written parental informed consents obtained.

Infants were excluded if they had:

1. Severe congenital malformations,
2. Cyanotic congenital heart disease,
3. Hereditary metabolic diseases,
4. Emergency intubation requirement after birth,
5. No parental informed consent.

### Surfactant administration

Calf Pulmonary Surfactant (Calsurf) was supplied in ready-to-use, single-use vials containing 70 mg of surfactant phospholipids. Calsurf is licensed through Beijing Double Crane Pharmaceutical CO., Beijing, China. Calsurf is a natural surfactant, prepared from bovine lungs, containing almost exclusively polar lipids, in particular phosphatidylcholine (≥ 55% of the total phospholipids) and 1–2% of surfactant associated apoproteins SP-B and SP-C. Dipalmitoyl-phosphatidylcholine, palmitic acid, and tripalmitin are added to standardize the composition and to mimic the surfactant properties of natural lung surfactant. Pulmonary surfactant dosage used was 70–140 mg/kg.

### The LISA + SNIPPV and InSurE + nCPAP technique

All infants were initially the same treatment strategy^19,20^. Without prior sedation or analgesia, the vocal cords were gently visualized using the laryngoscope and the thin, semi-rigid, narrow-bore tracheal catheter (Double Crane Pharmaceutical CO., Beijing, China) directly inserted under laryngoscopy, was carefully passed 1.5 to 2 cm beyond the cords without using Magill’s forceps. Calsurf was instilled into the tracheal catheter over 10 min by mini-boluses. Tracheal catheterization was only attempted by trained neonatal specialists, neither LISA nor InSurE use sedative drags. Respiratory support following the procedure was SNIPPV mode and breathing synchrony was achieved using an abdominal Graseby capsule placed on the subxiphoid region. Criteria for surfactant doses and management in InSurE + nCPAP group were the same as for the LISA group. Infants with RDS were immediately supported with nCPAP at 6–8 cmH_2_O and FiO_2_ was initially set at 0.21–0.4 and adjusted to obtain SpO_2_ of 90–94% by setting alarm limits. NIPPV group (peak inspiratory pressure 15–25 cm H_2_O, PEEP 4–6 cmH_2_O, inspiratory time 0.3–0.5 s, breathing frequency 15–40 breaths/min, FIO_2_ 0.21–0.40). After the child's condition is stabilised, the pressure can be gradually reduced, and CPAP can be considered to be discontinued when the pressure is < 4 ~ 5 cm H_2_O, FiO_2_ ≤ 0.25, there is no apnoea and bradycardia, TcSO_2_ does not fall, and the work of respiration does not increase. Similarly, when the child's condition is stabilised, all the parameters can be gradually reduced, and evacuation can be considered to be completed when the FiO_2_ is < 0.30, the peak inspiratory pressure (PIP) is < 14 cmH_2_O, PEEP < 4 cmH_2_O, respiratory rate < 15 breaths/min, the child has no apnoea, bradycardia, and TcSO_2_ has not decreased, the withdrawal of NIPPV can be considered^21^.

### Outcomes

#### Primary outcome

The primary outcome of the study was the rate of BPD^22^ and FiO_2_ requirements.

#### Secondary outcomes

The secondary outcomes analyzed included:

1. Mortality rate,
2. Duration of mechanical ventilation,
3. Duration of oxygen therapy,
4. Re-intubation rate,
5. Incidence of complications,
6. Electrolyte disturbances,
7. Acid–base disturbances,
8. Respiratory severity score (RSS)^23^: defined as FiO_2_ × (ventilatory support) + (medications), assessing the severity of neonatal lung disease and the risk of subsequent pulmonary morbidity,
9. Length of hospital stay.

### Statistical analysis

Statistical analysis of data was performed using SAS software, version 9.4. Comparison between the two groups was done using the chi-square test or Fisher exact test for categorical variables. Normally distributed continuous variables were compared by student's t-test; abnormally distributed continuous variables were compared by Wilcoxon rank sum test. A *P* value of < 0.05 was considered statistically significant.

### Ethics approval

The study was approved by The First Affiliated Hospital of Nanjing Medical University (number: 2018-SR-161). And We have registered the experiment with ClinicalTrails.gov, the registration number: NCT03989960 (Date:18/06/2019).

## Results

### Study population

The baseline characteristics of the study population are similar in both groups (Table 1).

**Table 1:** Baseline characteristics.

	LISA + SNIPPV (n = 32)	InSurE + CPAP (n = 57)	P-value
Birth weight, grams	1340.78 ± 325.41	1258.75 ± 323.54	0.26
Gestational age, weeks	29.50 ± 1.95	28.81 ± 1.61	0.07
Male gender	17 (53.13%)	36 (63.16%)	0.35
1-min Apgar score	6.88 ± 1.70	6.25 ± 2.49	0.16
5-min Apgar score	7.88 ± 1.60	7.61 ± 1.98	0.53
Premature rupture of membranes	9 (28.13%)	23 (40.35%)	0.25
Age of mother	30.50 ± 4.98	29.51 ± 5.28	0.39
Parity	1.72 ± 0.73	1.51 ± 0.63	0.16
Multiple births	8 (25.00%)	15 (26.32%)	0.89
Small for gestational age (SGA)	3 (9.38%)	10 (17.54%)	0.37
In vitro fertilization	5 (15.63%)	7 (12.28%)	0.75

### Primary outcome

**Figure 2:**
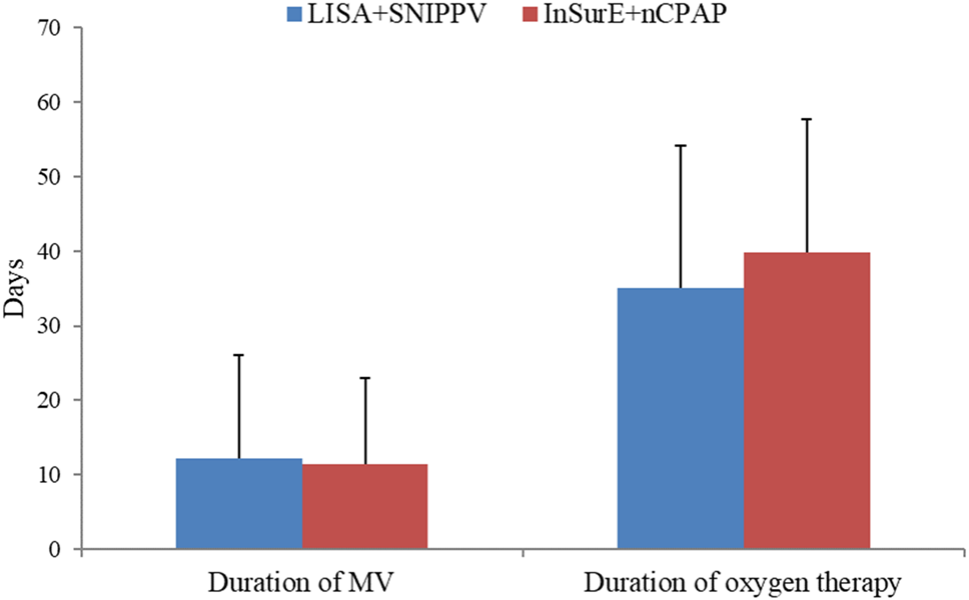
The differences in mean duration (days) of MV and oxygen therapy between the two study groups.

**Figure 3:**
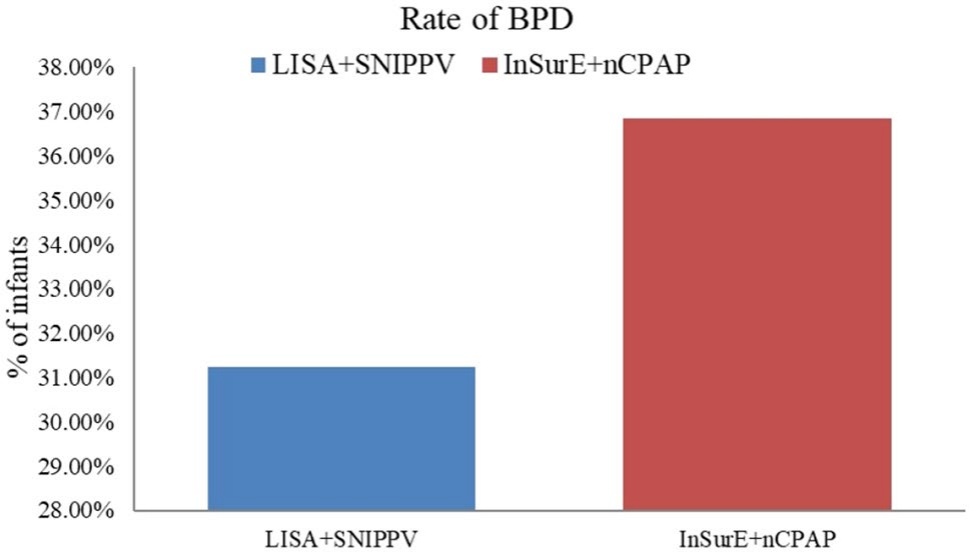
A lower BPD rate observed in the LISA + SNIPPV group compared to InSurE + nCPAP group; however the difference is not statistically significant.

FiO_2_ requirements were significantly lower in infants who underwent the LISA followed by SNIPPV up to the 3rd week after treatment [FiO_2_ at day 0, (30.75 ± 4.78) vs. (34.66 ± 9.83), P < 0.05; FiO_2_ at day 21, (25.32 ± 3.74) vs. (29.11 ± 8.17), P < 0.05] (Fig. 2). The rate of BPD was lower in the experimental group compared to the standard treatment group, without statistically significant [10 (31.25%) vs. 21 (36.84%), P > 0.05] (Table 2) (Fig. 3). The number needed to treat (NNT) with the combination of LISA + SNIPPV to prevent BPD development is 18.

**Table 2:** Rate of BPD, and secondary outcomes (durations of MV and oxygen therapy, re-intubation rate, incidence of complications, electrolytes and acid–base disturbances, length of hospital stay, mortality rate).

	LISA + SNIPPV (n = 32)	InSurE + CPAP (n = 57)	*P*-value
Primary outcome			
Rate of BPD	10 (31.25%)	21 (36.84%)	0.65
Secondary outcomes			
Duration of MV (days)	12.18 ± 13.89	11.35 ± 11.61	0.76
Duration of oxygen therapy (days)	35.03 ± 19.13	39.75 ± 17.91	0.25
Re-intubation rate	0.19 ± 0.40	0.21 ± 0.45	0.81
Length of hospital stay (days)	45.97 ± 16.93	54.40 ± 16.26	0.02*
Mortality rate	1 (3.13%)	2 (3.51%)	0.13
Complications			
Pneumothorax	0 (0.00%)	2(3.51%)	0.53
Pneumonia	18 (56.25%)	22 (38.60%)	0.11
Sepsis	4 (12.50%)	6 (10.53%)	0.74
Nosocomial infection	2 (6.25%)	2 (3.51%)	0.62
PDA	24 (75.00%)	27 (47.37%)	0.01*
PDA closure	15 (60.00%)	6 (20.69%)	0.01*
Cholestasis	4 (12.50%)	15 (26.32%)	0.13
Cerebral hemorrhage	20 (62.50%)	36 (63.16%)	0.95
NEC	3 (9.38%)	9 (15.79%)	0.53
Pulmonary hemorrhage	1 (3.13%)	2 (3.51%)	1.00
ROP	2 (6.45%)	5 (10.00%)	0.70
Apnea	25 (78.13%)	36 (63.16%)	0.14
PVL	1 (3.13%)	2 (3.51%)	1.00
Anemia	26 (81.25%)	54 (94.74%)	0.07
Hypoglycemia	9 (28.13%)	11 (19.30%)	0.34
Elevated transaminases	1 (3.23%)	19 (33.33%)	0.00*
Electrolyte disturbances			
Hypocalcemia	27 (84.38%)	39 (68.42%)	0.09
Hypophosphatemia	8 (25.00%)	6 (10.53%)	0.07
Hypokalemia	8 (25.00%)	8 (14.04%)	0.19
Hyperkalemia	9 (28.13%)	10 (17.54%)	0.24
Hypochloremia	6 (18.75%)	20 (35.09%)	0.10
Hypernatremia	4 (12.50%)	6 (10.53%)	0.74
Hypermagnesemia	12 (37.50%)	11 (19.30%)	0.05
Hypomagnesemia	19 (59.38%)	32 (56.14%)	0.76
Acid–base disturbances			
Respiratory acidosis	8 (25.00%)	49 (85.96%)	< 0.001*
Metabolic acidosis	5 (15.63%)	36 (63.16%)	< 0.001*
Respiratory alkalosis	0 (0.00%)	10 (17.54%)	0.01*
Metabolic alkalosis	0 (0.00%)	26 (45.61%)	< 0.001*

*Indicates significant differences with a *P* < 0.05.

### Secondary outcomes

**Figure 4:**
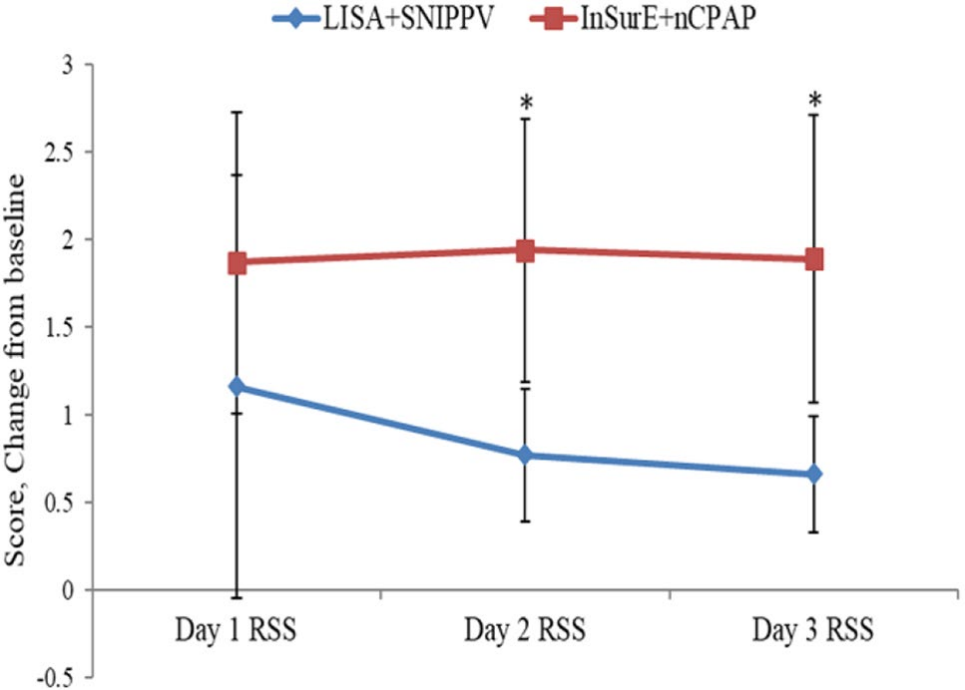
The graph illustrates the significantly lower RSS in the LISA + SNIPPV group on days 2 and 3 after treatment. *Indicates significant differences with a P < 0.05.

**Figure 5:**
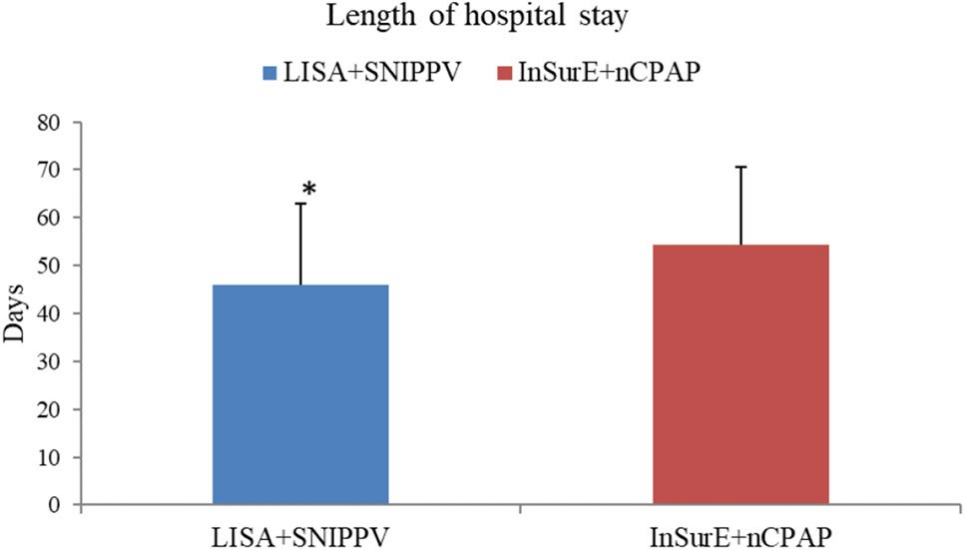
The graph illustrates the significantly prolonged length of hospitalization in the InSurE + nCPAP group compared to the experimental group. *Indicates significant differences with a *P* < 0.05.

**Figure 6:**
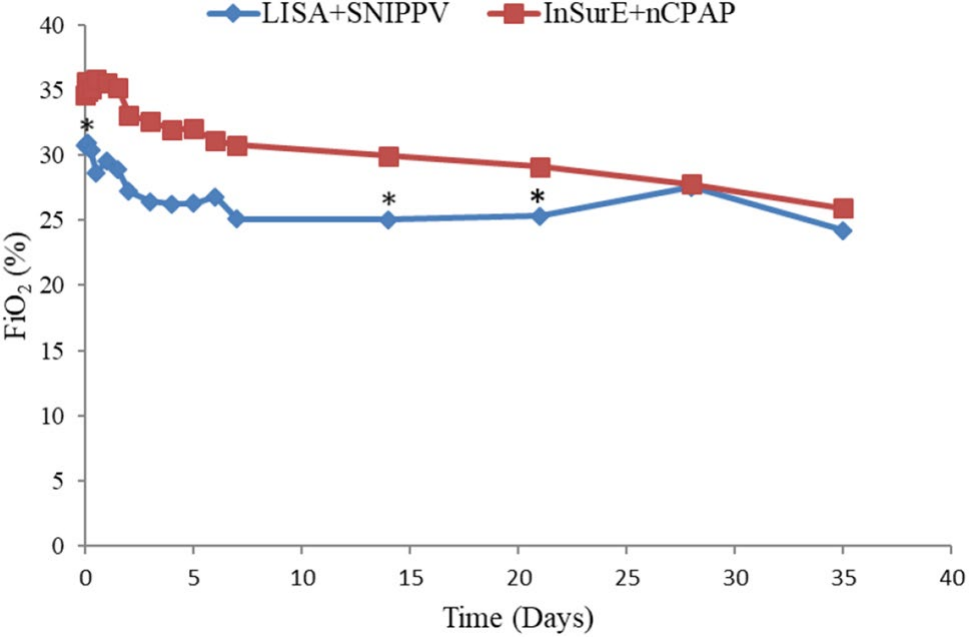
The blue line indicates infants receiving LISA + SNIPPV treatment and the red line indicates those receiving InSurE + nCPAP. The FiO_2_ requirement was significantly different immediately after treatment for both groups (At Day 0, mean FiO_2_ (%) for LISA + SNIPPV group was 30.75 ± 4.78 vs. 34.66 ± 9.83 for the standard group). A significantly lower FiO_2_ demand was sustained up to the 2rd (14 days) and 3rd week (21 days) after treatment (*P* < 0.05). *Indicates significant differences with a P < 0.05.

Mortality rates were not statistically different between the two study groups (Table 2). The duration of MV, oxygen therapy and re-intubation rates were statistically similar between the two study arms (Table 2) (Fig. 4). LISA-treated infants has a significantly lower RSS on days 2 [(0.77 ± 0.38) vs. (1.94 ± 0.75), P < 0.05] and 3 [(0.66 ± 0.33) vs. (1.89 ± 0.82), P < 0.05] after birth (Fig. 5). Analyzing complications incidences in the two groups revealed no statistically significant differences in complications. However, the LISA-treated group had a significantly higher rate of patent ductus arteriosus (PDA) and a higher rate of PDA closure (*P* < 0.05) and a significantly lower proportion of infants with disturbed liver function [1 (3.23%) vs. 19 (33.33%), P < 0.05] (Table 2). No intestinal perforation occurred in our study population. Furthermore, a significantly lower proportion of infants in the LISA group had acid–base disturbances and electrolyte disturbances (Table 2). The length of hospitalization was significantly lower for the LISA-treated infants compared to InSurE-treated ones [(45.97 ± 16.93) vs. (54.40 ± 16.26), P < 0.05] (Table 2) (Fig. 6).

## Discussion

In this era of high-quality perinatal and neonatal care, moving away from the traditional use of MV to administer surfactant and searching for the least invasive method of surfactant administration is crucial. While having numerous benefits compared to conventional MV, the InSurE technique still requires the use of an endotracheal tube and the damaging effects of short-term MV on the immature lungs have been demonstrated by animal models studies^24^. Transitioning from the use of an endotracheal tube to a nasogastric feeding tube or vascular catheter has revolutionized the strategy of surfactant administration and the LISA (nasogastric feeding tube) and MIST (vascular catheter) techniques have evolved to further minimize lung injury and prevent BPD^9,10^. The large Avoidance of Mechanical Ventilation (AMV) trial, investigating infants of 26 to 28 weeks’ GA, deducted that the LISA method lessens the need for MV^25^. Further investigation of LISA led to the Nonintubated Surfactant Application trial, which was conducted over three years and involved 13 level III NICU in Germany. It studied the application of LISA to preterm infants of 23 to 26 weeks’ GA with RDS, which was associated with reduced rates of intubation and a significantly lower incidence of major complications (pneumothorax and severe intraventricular hemorrhage); hence, emphasizing the sizeable impact on the infants’ quality of life^26^.

Apart from the method of surfactant administration, the post-extubation NIV support is another important concern to resolve. We combined LISA with SNIPPV as one of the goals of our study was to avoid CPAP failure. Despite the acclaimed positive effects of nCPAP such as reducing the need for and duration of MV and oxygen therapy, the use of nCPAP may not be sufficient in patients with severe surfactant deficiency^26–28^. Failure of CPAP support is an incontestable weakness of the CPAP technique, which occurs in more than 50% of extremely premature infants^29^. CPAP failure can be predicted in infants supported by CPAP in the initial hours after birth by an FiO2 exceeding 0.30. Some studies have also reported predictive factors of CPAP failure, including gestational age, male gender, birth weight, Apgar score at 1 or 5 min and oxygenation parameters^29–31^.

Evidence from a previous study comparing nCPAP to NIPPV during the MIST procedure found NIPPV to be more favorable for preterm infants with RDS^32^. Compared to nCPAP, NIPPV reduces the risk of extubation failure and the need for re-intubation, but has no effect on chronic lung disease and mortality^33^. As described above, the benefits of NIPPV over nCPAP have been well-acknowledged in literature. Synchronization further amplifies the positive effects of NIPPV and as reported by Huang et al. it improves gas exchange and reduces respiratory effort after extubation^34^. Moreover, SNIPPV compared to CPAP after the INSURE procedure was found to decrease the need for MV^15^.

With the aim of reinforcing the currently existing evidence surrounding the LISA technique, our study evaluated the combination of the novel LISA technique and SNIPPV compared to InSurE and nCPAP. To improve the odds of initial successful catheterization and reduce the operation duration and incidence of adverse effects (bradycardia and desaturations) during LISA, we chose to use a semi-rigid, narrow-bore tracheal catheter that could be directly inserted under laryngoscopy, without using Magill’s forceps. As shown in Table 1, our two study groups had no differences in their baseline characteristics and all infants were initially management^19^. The therapeutic plan only differed based on the surfactant administration method and the post-extubation mode of non-invasive respiratory support. In our study center, tracheal catheterization was only attempted by trained neonatal specialists and owing to the simplicity of the LISA technique, the trachea was successfully catheterized at the first attempt for all infants in the LISA group. In relation to airway manipulation with the laryngoscope blade, occasional bradycardic and desaturation events were witnessed, but were self-limited and resolved within a few seconds without resorting to the use of positive pressure ventilation. Surfactant administration via the LISA catheter was followed by an instantaneous physiologic effect, demonstrated by a rapid reduction in FiO2 requirement.

Analysis of the primary outcome of our study revealed a lower rate of BPD development in the experimental group, but it did not differ significantly from the traditional InSurE group. This might be due to our relatively small sample size, which requires expansion in the near future to fully assess the impact of LISA + SNIPPV in terms of BPD development. Secondary outcomes analysis found no statistically significant differences in the duration of MV and oxygen therapy and re-intubation rates for LISA + SNIPPV-treated and for InSurE + nCPAP-treated infants. These results should be interpreted with caution due to our small study sample. Moreover, as illustrated in Fig. 6, the significantly lower FiO2 values for the LISA-treated infants compared to the InSurE-treated ones over the first 3 weeks after treatment shows that lower concentrations of oxygen is required by infants following the LISA method. This finding is favorable for premature infants to reduce the risks of hyperoxia and oxidative stress and may be the reason for the tendency of lower BPD rate in the LISA group^10^. Moreover, the RSS differed significantly on days 2 and 3 between the two groups; the lower RSS in the experimental group is also related to the lower FiO2 requirements in the LISA group, and it indicates less lung damage and a lower risk of subsequent pulmonary morbidity^23^. As noted in a previous study, the higher incidence of PDA observed in our LISA-treated infants was possibly due to the left-to-right shunt created by the cascade effect following a decrease in pulmonary vascular resistance following surfactant administration^32^. However, potential improvement in lung compliance and lowered pulmonary artery pressure with surfactant therapy did not increase the poor prognosis associated with hemodynamically-significant PDA and a higher rate of PDA closure subsequent to oral ibuprofen treatment was witnessed in our experimental group. Although some previous studies have reported a higher incidence of gastrointestinal complications associated with NIPPV, we did not observe similar trends^33^. NIPPV did not increase the incidence of gastrointestinal complications in the LISA + SNIPPV group and a possible rationale is the role of synchronization, which increases comfort and reduces flatulence^35^. Similarly, we found normal growth in both groups of preterm infants, neither of which reached the diagnosis of extrauterine growth retardation, which could also be due to our small sample size. Laboratory indexes analysis revealed significantly lower incidences of acid–base disturbances in the LISA-treated group. The latter was also reported by Moretti et al. whereby respiratory acidosis was not a factor for extubation failure in infants treated with SNIPPV^36^. They concluded that SNIPPV could better stimulate the respiratory drive. The incidence of elevated transaminases was lower in the intervention group and in terms of electrolyte disturbances, there were no statistically significant differences between the two study groups. Consequently, the LISA procedure can be considered safe and well-tolerated. Furthermore, LISA-treated infants also had the added benefit of considerably fewer hospitalization days compared to the standard treatment group. Hence, the experimental group demonstrated improved, short-term clinical outcome, which may significantly improve the prognosis of RDS infants.

Previous larger studies have reported a lower incidence of BPD development in infants treated with LISA^37–40^. In 2010, the first multicentre report for LISA was published by Kribs et al. which showed significantly decreasing need for MV in the first 72 h, incidence of BPD (10.9% vs. 17.5%, P = 0.004) and mortality rates in the LISA group compared to the standard care group^37^. Additionally, higher prevalence of surfactant, theophylline, caffeine and doxapram use and less frequent use of analgesics, catecholamines and steroids were observed in the LISA group. In their randomized study, Kanmaz et al. compared the Take Care (LISA) and InSurE methods in preterm infants < 32 weeks^38^. They not only found a reduced need for and duration of MV, but also a significantly lower rate of BPD, for infants treated with the less invasive technique of surfactant administration. A study in Poland also found similar results for BPD with the use of LISA^39^. Additionally, a large cohort study found LISA to be associated with a significant reduction in invasive ventilation (12% versus 18%, P = 0.001), reduced postnatal steroid use (2.5% versus 7%, P < 0.001), BPD (12% versus 18%, P = 0.001) and BPD or death (14% versus 21%, P < 0.001)^40^. Furthermore, Wu et al. analyzed the results from selected trials using LISA and validated the beneficial aspects of the thin-catheter strategy in terms of decreased requirement of MV and, consequently, a reduction in BPD incidence^41^.

On the other hand, similar to our findings, the effect of LISA was comparable to standard care in some studies^42–44^. A recently published RCT by Gupta et al. compared the MIST and InSurE techniques for the treatment of RDS in preterm infants of < 34 weeks GA, with NIPPV used as primary respiratory support^44^. They found no differences between the two study groups in terms of need for invasive MV in the first 72 h of life, hsPDA, IVH, BPD or the composite outcome of BPD and death. However they also reported a longer hospital stay for InSurE-treated infants. The reasons for dissimilar proposed conclusions are multi-factorial. The studied sample size, criteria for patient selection, patient demographics and study designs differ among the studies and have an impact on the final inferences. For widespread establishment of the LISA technique in clinical settings, larger randomized trials are warranted to decipher the optimal combination of the type of catheter and NIV support to be used and to address the issue of premedication.

The surfactant preparation used in our study was Calsurf as it has been carefully evaluated in clinical trials since 1997. It has proven to be an excellent candidate for acute and temporary replacement of pulmonary surfactant in infants with RDS^45^. Calsurf treatment has been reported to reduce the incidence of neonatal mortality in babies with RDS. Meta-analysis demonstrated that modified bovine minced lung surfactant extract was inferior to the porcine minced lung surfactant extract in terms of the risk of mortality prior to hospital discharge, death or oxygen requirement at 36 weeks’ PMA, repeated surfactant doses, etc^46^. However, no difference in outcome was noted between bovine lung lavage surfactant extract versus porcine minced lung surfactant extract or porcine lung lavage surfactant. A comparative effectiveness study of three surfactant preparations (calfactant, beractant and poractant) demonstrated similar effectiveness in the prevention of air leak syndromes, death, and BPD or death in premature infants^47^. Compared with Curosurf, Calsurf reduced in-hospital costs but had similar effects on treatment of RDS in full-term and preterm neonates^48^.

## Limitations

Firstly, it was a single-center study and our sample size could be further expanded to obtain results more representative of the population. Secondly, allocation of participants to treatment interventions was not randomized and although blinding was abided by for data analysis, operators could not be blinded to the surfactant administration strategies. Thirdly, it is important to acquire further data by long-term follow-up of discharged patients to gain better insight of RDS infants.

## Conclusions

Our research focused on the innovative LISA and SNIPPV, a superior mode of NIV support compared to CPAP. Our study showed a trend of lower rate of BPD in infants treated with LISA followed by SNIPPV. Moreover, preterm infants treated by the LISA + SNIPPV would undeniably positively benefit from the lower RSS, shorter hospitalization and lower FiO_2_ demands, better prognosis and an enhanced quality of life with a reduced risk of adverse outcomes related to hyperoxia and oxidative stress which are established high-risk factors of BPD. However, Validation of our results warrants a larger sample-sized and multi-centered clinical study for further research.

## Data Availability

The data can be available upon request from the corresponding author, Dr. Xiao-qing Chen.
