# Author Correction: A clinical study evaluating the combination of LISA and SNIPPV for the treatment of respiratory distress syndrome in preterm infants

**DOI:** 10.1038/s41598-024-55787-y

**Published:** 2024-03-04

**Authors:** Dhivya Lakshmi Permall, Yuhan Zhang, Hanyue Li, Yafei Guan, Xiaoqing Chen

**Affiliations:** 1 Nanjing Medical University, Nanjing, Jiangsu China; 2 Department of Pediatrics, The First Affiliated Hospital of Nanjing Medical University, Nanjing, Jiangsu China

Correction to: *Scientific Reports* 10.1038/s41598-023-50303-0, published online 16 January 2024

In the original version of this Article Dhivya Lakshmi Permall and Yuhan Zhang were omitted as equally contributing authors.

The original Article has been corrected.

